# P2Y_2_receptor activation by nucleotides released from highly metastatic breast cancer cells increases tumor growth and invasion via crosstalk with endothelial cells

**DOI:** 10.1186/bcr3694

**Published:** 2014-08-26

**Authors:** Hana Jin, So Young Eun, Jong Sil Lee, Sang Won Park, Jae Heun Lee, Ki Churl Chang, Hye Jung Kim

**Affiliations:** 1 Department of Pharmacology, School of Medicine, Institute of Health Sciences, Gyeongsang National University, Jinju, Korea; 2 Department of Pathology, School of Medicine, Institute of Health Sciences, Gyeongsang National University, Jinju, 660-751 Korea

## Abstract

**Introduction:**

Extracellular nucleotides are released and detectable in a high concentration within the tumor microenvironment. G protein-coupled P2Y_2_ nucleotide receptor (P2Y_2_R) is activated equipotently by adenosine triphosphate (ATP) and uridine 5′-triphosphate (UTP), which mediate proinflammatory responses such as cell migration and proliferation. However, the role of P2Y_2_R in the process of cancer metastasis remains unclear. This study aimed to determine the role of P2Y_2_R in the proliferation, migration and invasion of highly metastatic MDA-MB-231 breast cancer cells through crosstalk with endothelial cells (ECs).

**Methods:**

ATP release and P2Y_2_R activity between high metastatic breast cancer cell MDA-MB-231 and low metastatic breast cancer cell MCF-7 were compared. Then, the role of P2Y_2_R on tumor growth and invasion via crosstalk with ECs was examined in vitro, using MDA-MB-231 cells and ECs transfected with control- or P2Y_2_R-siRNA, and in vivo, using an animal model injected with control-shRNA- or P2Y_2_R-shRNA-transfected MDA-MB-231 cells.

**Results:**

We found that this highly metastatic breast cancer cell line released higher levels of ATP and showed a higher P2Y_2_R activity in comparison to a low metastatic breast cancer cell line, MCF-7. In MDA-MB-231 cells, P2Y_2_R activation by ATP or UTP increased proliferation at 24 or 72 hours, which was abolished by P2Y_2_R knock-down. In addition, the adhesion of MDA-MB-231 cells to ECs and cell migration were both significantly increased by ATP or UTP through the expression of intercellular adhesion molecule-1 (ICAM-1) and vascular cell adhesion molecule-1 (VCAM-1) in MDA-MB-231 or ECs but not in cells where P2Y_2_R was knocked down. Furthermore, ATP- or UTP-mediated activation of P2Y_2_R induced MDA-MB-231 invasion through ECs, increased matrix metalloproteinase-9 (MMP-9) activity and vascular endothelial growth factor (VEGF) production in MDA-MB-231 and induced the phosphorylation of vascular endothelial (VE)-cadherin in ECs. Tumor growth and metastasis to other tissues were dramatically reduced, and body weight was increased in mice injected with P2Y_2_R-shRNA-transfected MDA-MB-231 cells compared to mice injected with control shRNA-transfected MDA-MB-231 cells.

**Conclusion:**

This study suggests that P2Y_2_R may play an important role in cancer metastasis via modulation of the crosstalk between cancer cells and ECs.

**Electronic supplementary material:**

The online version of this article (doi:10.1186/bcr3694) contains supplementary material, which is available to authorized users.

## Introduction

Nucleotides, especially ATP, were long thought to be restricted to the intracellular compartment, where they are involved in biochemical reactions such as energy transactions and nucleic acid synthesis. However, nucleotides were recently shown to be released from injured or stressed cells and tissues [1–4] and to mediate various cellular responses through activation of cell surface P2 receptors, G protein-coupled P2Y receptors (P2YRs) and ion channel P2X receptors (P2XRs). ATP is also released and accumulates in a much higher concentration in the tumor interstitium than in healthy tissues [5], and recent reports have highlighted the involvement of ATP in tumor progression. Depending on the dose and the purinergic P2 receptor subtype engaged, ATP can trigger many different cellular responses, ranging from cell death to proliferation [6–8]. Among the receptors engaged by extracellular ATP (P2 receptors), the P2Y_2_R is most consistently expressed (or overexpressed) by tumor cells, and it mediates proliferation in many tumors, for example, melanoma [9], lung [10], bladder [11] and prostate cancer [12]; however, controversial phenomena have also been reported in other tumor types, for example, esophageal [13], colorectal [14] and ovarian cancer [15]. Thus, further study is needed to determine how P2Y_2_R affects tumor progression depending on cancer cell type and the conditions of the tumor microenvironment.

Breast cancer is the most common cancer diagnosed in North American and Western European women [16, 17], and Asian populations generally have the lowest risk, but rates in this population have been steadily increasing. Virtually all patients who die from breast cancer have metastatic disease. Cancer metastasis is a complex process involving the coordinated cellular responses of both cancer cells and normal cells; invasion of the stroma, intravasation of the blood vessel, circulation in the blood, lodging and adhesion in the target capillaries, extravasation from the blood vessels and proliferation of secondary tumors [18].

Several cell adhesion molecules (CAMs), including intercellular adhesion molecule-1 (ICAM-1) and vascular cell adhesion molecule-1 (VCAM-1) have been implicated in cancer growth, and metastasis [19, 20]. CAMs are expressed on a variety of cells, including vascular endothelial cells (ECs), lymphocytes, fibroblasts, hematopoietic cells and tumor cells [21–24]. Some CAMs, such as VCAM-1, are expressed preferentially or at higher levels on the breast cancer endothelium compared to the normal endothelium [21, 25]. ICAM-1 and VCAM-1 are involved in cell-cell and cell-extracellular matrix (ECM) interactions, and they are mechanistically important for the extravasation of monocytes during inflammation [26] and cancer cells during metastasis [27, 28]. Therefore, the adhesion of circulating tumor cells to the microvascular endothelium of organs at distant sites, such as the liver and lungs, is an important step in blood-borne metastasis. Another important molecule that is critical for tumor metastasis is the transmembrane endothelial adherens junction (AJ). In ECs, AJs are largely composed of vascular endothelial cadherin (VE-cadherin), an endothelium-specific member of the cadherin family of adhesion proteins that binds, via its cytoplasmic domain, to several protein partners such as p120, β-catenin and plakoglobin [29]. Thus, we were specifically interested in the role of P2Y_2_R in cancer metastasis via the regulation of adhesion molecules (AMs) and VE-cadherin.

Estrogen receptor (ER)-positive (ER+) breast cancers generally have a better prognosis and are responsive to anti-estrogen therapy. In contrast, ER-independent (ER-) breast cancers are more aggressive, possess high metastatic potential and are unresponsive to anti-estrogens [30, 31]. As tumor metastasis is considered the main cause of mortality in cancer patients, it is beneficial to study how ER- human MDA-MB-231 breast cancer cells metastasize. In this study, we hypothesized that conditions of the tumor microenvironment, specifically the high level of ATP released from cancer cells, may affect tumor progression and metastasis via crosstalk with ECs. Preliminary data revealed that MDA-MD-231 cells release ATP at a much higher level than MCF-7 cells; therefore, in this study, we investigated the role of P2Y_2_R in cancer metastasis through crosstalk with ECs using the breast cancer cell line MDA-MB-231.

## Methods

### Cell culture

The human breast cancer cell lines MCF-7 and MDA-MB-231 were obtained from the Korea Cell Line Bank (Seoul, Korea), and the human umbilical vascular endothelial cell line EA.hy 926 and the spontaneously immortalized human normal breast epithelial cell line MCF10A were originally purchased from the American Type Culture Collection ATCC, Vanassas, VA, USA). MCF-7 and MDA-MB-231 were grown in RPMI 1640 supplemented with 10% FBS, 100 IU/ml penicillin and 10 μg/ml streptomycin, and MCF10A was grown in DMEM/F-12 medium supplemented with 5% horse serum, 100 U/ml of penicillin, 100 g/ml of streptomycin, 0.5 g/ml hydrocortisone, 100 ng/ml cholera toxin, 10 g/ml insulin, 10 ng/ml epidermal growth factor and 1% (w/v) of L-glutamine. EA.hy 926 was grown in DMEM supplemented with 10% FBS, 100 IU/ml penicillin and 10 μg/ml streptomycin.

### Extracellular ATP release measurements

Cells were incubated for 15 minutes at 37°C with HEPES buffer (pH 7.4) containing AOPCP, a selective inhibitor of ecto-5′-nucleotidase. Cells were treated with or without TNF-α for an additional 5 minutes. Supernatants were collected at specific time points, and ATP release was measured with the ENLITEN ATP assay system kit (Promega, Madison, WI, USA). ATP levels were calculated based on an ATP standard curve.

### Gene silencing with small interferin RNA (siRNA) or small hairpin RNA (shRNA)

Gene silencing experiments were performed with three independent P2Y_2_R-siRNA or shRNA. Cells were transfected with 100 nM control (CTRL) siRNA/P2Y_2_R siRNA (Bioneer, Daejeon, Korea) or 10 μg/ml of shRNA (Santa Cruz; Santa Cruz, CA, USA) in serum-containing medium using Turbofect® (Thermo Scientific, Rockford, IL, USA). Gene silencing efficiency was determined by reverse transcription-polymerase chain reaction (RT-PCR) and western blot analysis.

### RT-PCR

RT-PCR was performed using TOPscript One-step RT PCR Drymix (Enzynomics, Daejeon, Korea), according to the manufacturer’s instructions. The primer sets used were as follows: hP2Y_2_R, 5′-GTG CTC TAC TTC CTG GCT-3′ and 5′-CTG AAG TGT TCT GCT CCT AC-3′ and hGAPDH, 5′- TCA ACA GCG ACA CCC ACT CC-3′ and 5′- TGA GGT CCA CCA CCC TGT TG-3′.

### Cell proliferation assay

Cells at the exponential growth phase were seeded at 10^4^ cells per well in 24-well plates. After the indicated treatments, the cells were washed with ice-cold PBS, harvested and mixed with a 0.4% trypan blue solution. Viable cells in the cell suspension were counted with a hemacytometer under a light microscope.

### Western blot analysis

Western blot analysis was performed as described previously [32], with minor modifications. Briefly, aliquots of 50 μg of protein were subjected to 7.5% SDS-PAGE and transferred onto Hybond-P + polyvinylidene difluoride membranes (Amersham Biosciences UK Ltd). The membranes were incubated with primary antibodies; anti-ICAM-1, -VCAM-1, -P2Y_2_R (Santa Cruz) or -phosphor-VE-cadherin (Y658) (Abcam, Cambridge, UK). β-Actin (Sigma Aldrich, St Louis, MO, USA) was used as loading control for normalization to the protein expression.

### Measurement of intracellular calcium ion concentration

The calcium ion ((Ca^2+^)_i_) concentration was measured as described previously [33]. Briefly, cells were stained with 5 μM fluo-3-AM and washed with physiological solution (125 mM NaCl, 5 mM KCl, 1 mM MgCl_2_, 10 mM HEPES, 5 mM glucose, and 1 mM CaCl_2_). Then, cells were treated with ATP or uridine 5′-triphosphate (UTP) and fluorescent images were scanned every 5 sec using a confocal microscope (IX70 Fluoview, Olympus, Tokyo, Japan; excitation wavelength 488 nm, emission wavelength 530 nm). The changes in (Ca^2+^)_i_ were calculated as follows:

$$\text{Change}\;\text{in}\;{\left({\text{Ca}}^{2+}\right)}_{i}=\left(F_{\text{max}}‒F_{0}\right)/F_{0}$$

With F representing the fluorescence intensity; F_0,_ the basal fluorescence intensity before treatment, and F_max_, the maximum level of fluorescence intensity, which occurred after the addition of ATP or UTP).

### Adhesion assay

ECs and MDA-MB-231 cells were transfected with CTRL siRNA or P2Y_2_R siRNA, as described above. The transfected cells were treated with 10 μM of ATP or UTP for 6 h. ECs were washed three times with fresh serum-free medium, and MDA-MB-231 cells (7.5 × 10^5^ cells/ml) were added to the ECs. After 30 minutes at 37°C, cell suspensions were withdrawn, and the ECs were gently washed with PBS three times. The cells were then counted under a light microscope, and the number of adhesive cells on ECs was quantified.

### Migration assay

MDA-MB-231 cells were transfected with CTRL siRNA or P2Y_2_R siRNA and treated with 10 μM ATP or UTP for 6 h. Then, 2 × 10^5^ cells were added to the upper chambers of the inserts, which were placed into a 24-well plate, and 500 μl of RPMI medium was added to the lower chambers. The migration chambers were incubated for 24 h in a 37°C cell culture incubator. The non-migrating cells that remained on the upper surface of the insert membranes were removed by scrubbing. The cells that had migrated across the insert-well membrane were stained with 4′,6-diamidino-2-phenylindole dihydrochloride (DAPI), and the cells were counted under a fluorescence microscope. Each experiment was repeated three times in triplicate.

### Matrigel invasion assay

For invasion assays, the upper chambers of inserts were coated with 100 μl of Matrigel (1 mg/ml, BD Bioscience, San Jose, CA, USA), and ECs (2 × 10^5^ cells) were added to the Matrigel-coated insert wells. MDA-MB-231 cells (2 × 10^5^ cells per insert) were added to the upper chambers in serum-free medium, and 500 μl of RPMI medium was added to the lower chambers. The rest of the procedure was carried out as described for the migration assay.

### Gelatin zymography

Media were concentrated 20-fold using protein concentrators (9 K MWCO, Thermo Pierce, Rockford, IL, USA) and subjected to electrophoresis on 8% PAGE gels containing 1 mg/ml gelatin. Gels were washed twice with 2.5% Triton X-100, stained with 0.2% Coomassie Brilliant Blue and de-stained (50% methanol and 10% acetic acid).

### Quantitative human vascular endothelial growth factor (VEGF) immunoassay

The level of VEGF in the conditioned medium from MDA-MB-231 cells was determined using a VEGF enzyme-linked immunosorbent assay kit (R&D Systems, Minneapolis, MN, USA), according to the manufacturer’s instruction. All assays were performed on triplicate plates.

### Animal experiments

We stably transfected MDA-MB-231 cells with expression vectors encoding shRNAs targeting P2Y_2_R (MDA-MB-231-P2Y_2_R-shRNA) or with an empty vector (MDA-MB-231-EV). Athymic nude mice were divided into two groups and injected subcutaneously with empty vector-transfected MDA-MB-231 (MDA-MB-231-EV; n = 10) or P2Y_2_R-shRNA-transfected MDA-MB-231 (MDA-MB-231-P2Y_2_R-shRNA; n = 10) (5 × 10^6^ cells/100 μl of serum-free medium). The experimental protocol was approved by the Institutional Animal Care and Use Committee at Gyeongsang National University (approval number: GLA-120208-M004). Body weights and tumor volumes were measured every 3 days, starting at 7 days after injection. At the end of 60 days, the mice were sacrificed. Lung tissue or tumor mass was fixed in 4% paraformaldehyde at room temperature, followed by paraffin infiltration and embedding. Sections of 5 μm were mounted onto ProbeOn Plus microscope slides (Fisher Scientific, Loughborough, UK) and stained with H&E. Immunohistochemical analysis was performed using anti-vimentin, -VCAM-1, -VEGF, -ICAM-1 and CD31 antibodies for lung staining, and H&E and anti-P2Y_2_R antibody (Abcam) for tumor staining, and the staining was examined under a light microscope.

### Statistical evaluations

Scanning densitometry was performed using Image Master® VDS (Pharmacia Biotech Inc, San Francisco, CA, USA). The treatment groups were compared using one-way analysis of variance and the Scheffe post-hoc test. All data were expressed as the mean ± standard error of the mean (SEM). Statistical analysis of the frequency of lung metastasis in the two animal groups was performed by Fisher’s exact test. *P* <0.05 was considered statistically significant.

## Results

### MDA-MB-231 highly metastatic breast cancer cells release higher levels of ATP and show a higher P2Y_2_R activity than the low metastatic breast cancer cell line MCF-7 or normal cells

**Figure 1:**
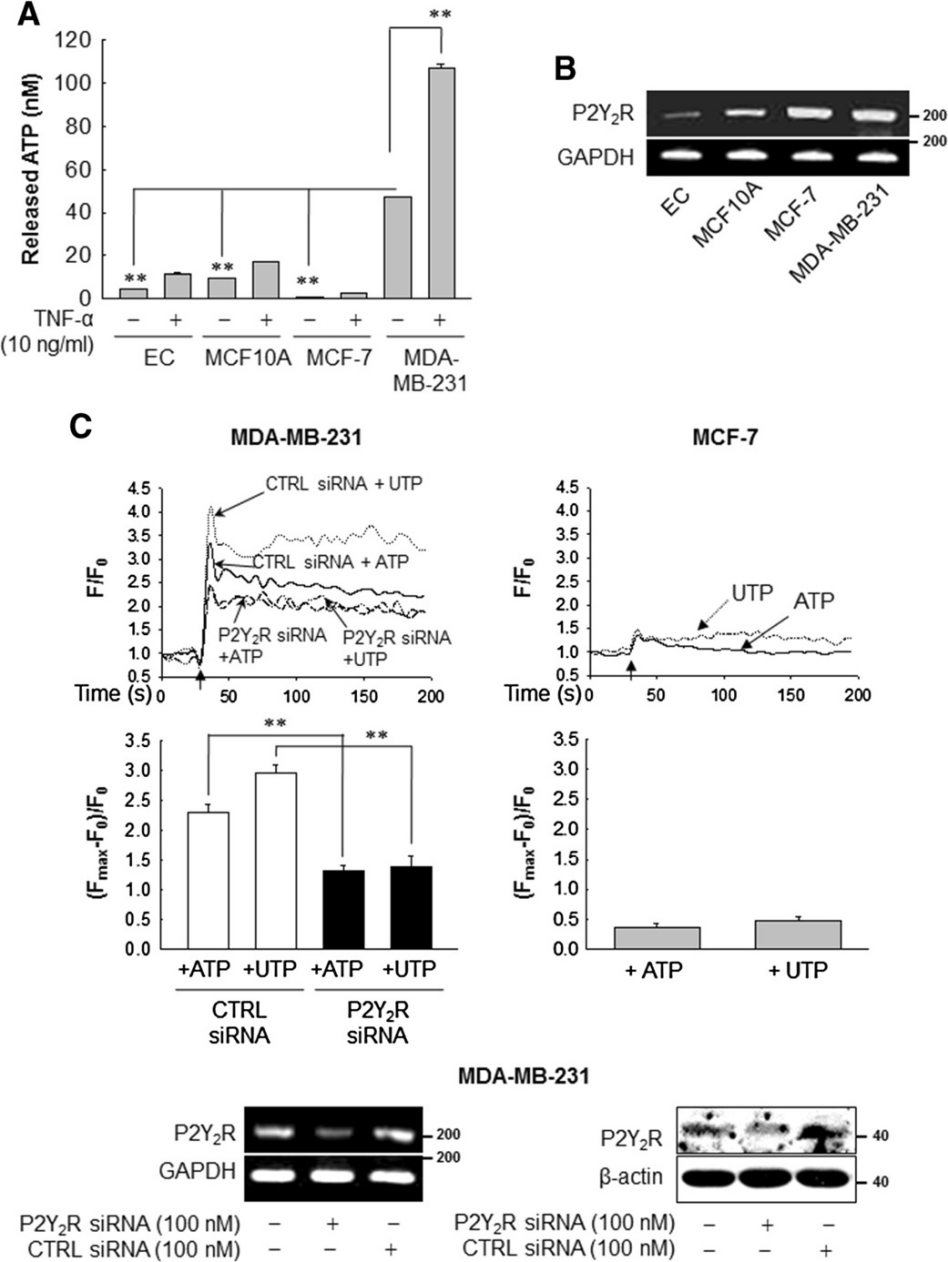
**ATP release and P2Y**_**2**_**R expression and activity in various cell types. (A)** The amount of ATP released into the extracellular medium was measured as described in Methods. Significance compared to MDA-MB-231, **P <0.01. **(B)** Total RNA was collected from endothelial cells (ECs), MCF10A, MCF-7 and MDA-MB-231, and P2Y_2_R (200 bp) and glyceraldehyde-3-phosphate dehydrogenase (GAPDH) (125 bp) mRNA expression was analyzed by RT-PCR. The results were confirmed by at least two independent experiments. **(C)** Intracellular Ca^2+^ levels were determined in MDA-MB-231 and MCF-7 to measure the P2Y_2_R activity. Arrows indicate the points at which ATP or uridine 5′-triphosphate (UTP) (10 μM) was added. The net change in Ca^2+^ levels was normalized to (F_max_-F_0_)/F_0_. Significance compared to ATP or UTP, **P <0.01.

First, we compared the levels of ATP released into the extracellular medium by various cell types. In normal conditions, the highly metastatic breast cancer cell line MDA-MB-231 released markedly more ATP in comparison to ECs, MCF10A (normal breast epithelial cells) and MCF-7 (low metastatic breast cancer cell). In addition, TNF-α, an essential factor in tumor progression and metastasis [34, 35], significantly enhanced the release of ATP, especially in MDA-MB-231 (Figure 1A). Moreover, RT-PCR revealed that P2Y_2_R mRNA was present in ECs, MCF10A, MCF7 and MDA-MB-231. Interestingly, P2Y_2_R mRNA levels were higher in the MCF-7 and MDA-MB-231 as compared to normal ECs or MCF-10A, and there was no significant difference between P2Y_2_R mRNA expression in MCF-7 and MDA-MB-231 (Figure 1B). To further compare P2Y_2_R activity between MCF-7 and MDA-MB-231, we measured the intracellular Ca^2+^ level (Ca^2+^)_i_ in response to agonist ATP or UTP. ATP or UTP (10 μM) elicited the immediate and rapid augmentation in (Ca^2+^)_i_ in MDA-MB-231, which was significantly reduced in P2Y_2_R-knocked-down MDA-MB-231. Interestingly, the transient elevation of (Ca^2+^)_i_ levels in MCF-7 were much lower than MDA-MB-231 (Figure 1C), suggesting the difference in P2Y_2_R activity in response to nucleotides between MCF-7 and MDA-MB-231.

### P2Y_2_R activation by ATP or UTP increases proliferation, migration and expression of adhesion molecules in MDA-MB-231 cells

**Figure 2:**
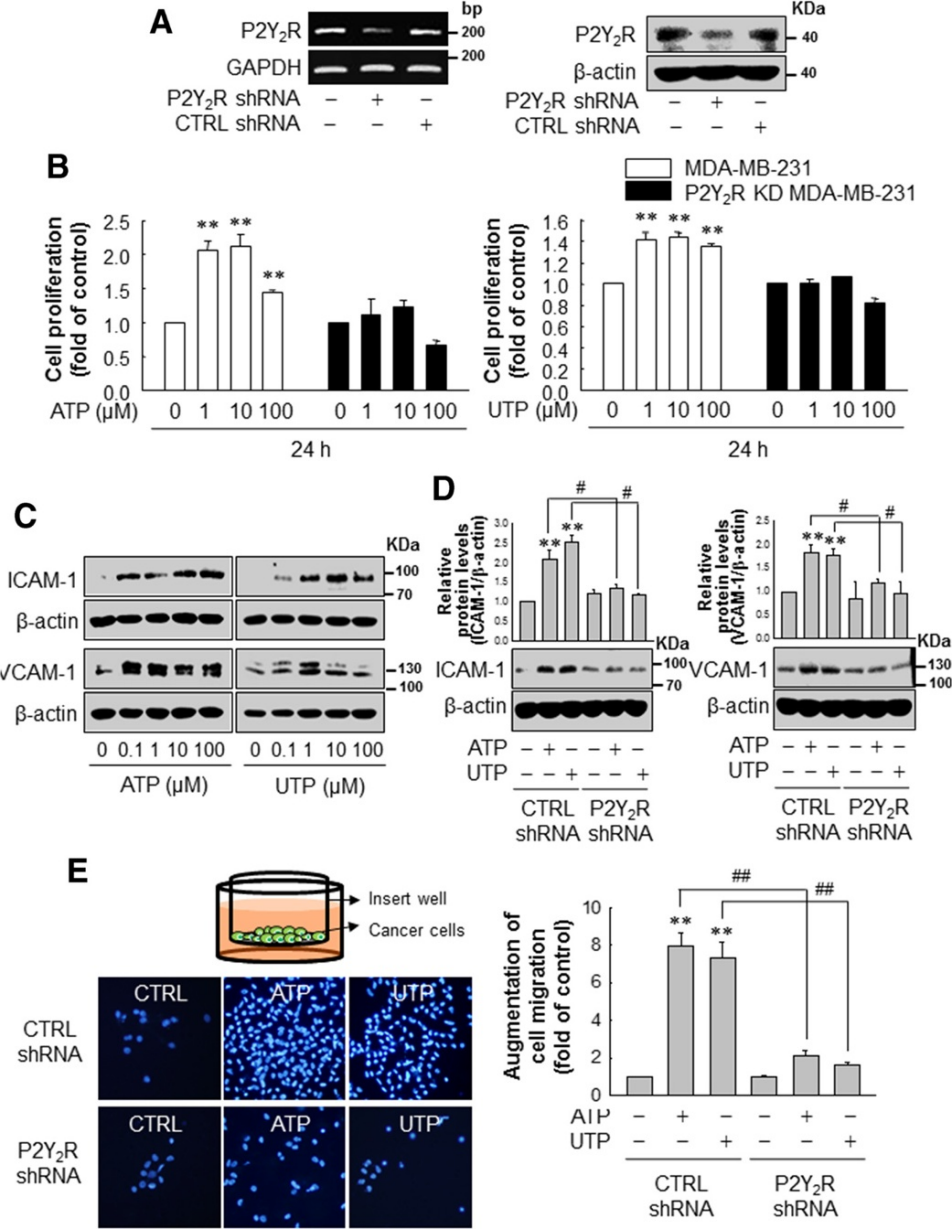
**P2Y**_**2**_**R activation by ATP or UTP induced MDA-MB-231 cell proliferation, migration and expression of adhesion molecules. (A, B)** Control- or P2Y_2_R-shRNA-transfected MDA-MB-231 were treated with various concentrations of ATP or UTP, as indicated. After 24 h, cell proliferation was determined by trypan blue exclusion assay. Significance compared to the control, **P <0.01. **(C)** MDA-MB-231 were treated with the indicated doses of ATP or UTP for 6 h. ICAM-1, VCAM-1 and β-actin expression levels were analyzed by western blotting. **(D)** Control- or P2Y_2_R-shRNA-transfected MDA-MB-231 were treated with ATP or UTP (10 μM) for 6 h, and ICAM-1 (88 to 110 KDa) and VCAM-1 (130 KDa) expression levels were determined as described previously. Significance compared to the control, **P <0.01; significance compared to ATP or UTP, ^#^*P* <0.05. **(E)** Control- or P2Y_2_R-shRNA-transfected MDA-MB-231 were treated with ATP or UTP (10 μM). Six hour later, the cells were harvested, and seeded onto cell culture inserts. After 24 h, the cancer cells that had migrated across the insert well membrane were stained with DAPI, and the number of migrated cells was counted under a fluorescence microscope and quantified. Significance compared to the control, **P <0.01; significance compared to ATP or UTP, ^##^*P* <0.01.

We transfected MDA-MB-231 with scrambled RNA or P2Y_2_R shRNA to elucidate the role of P2Y_2_R in the proliferation of breast cancer cells. After confirming the efficiency of P2Y_2_R shRNA at the mRNA and protein levels (Figure 2A), cells were treated with ATP or UTP (1, 10, 100 μM) for 24 h. P2Y_2_R activation by ATP or UTP significantly increased MDA-MB-231 proliferation at a low dose (1 μM), whereas the proliferation of P2Y_2_R-shRNA-transfected MDA-MB-231 was not affected by treatment with ATP or UTP (Figure 2B). In addition, we assessed the effect of P2Y_2_R on ICAM-1 and VCAM-1 expression after stimulating MDA-MB-231 with ATP or UTP and found that both ATP and UTP upregulated the expression of ICAM-1 and VCAM-1 at the indicated doses (Figure 2C), whereas the expression of ICAM-1 and VCAM-1 stimulated by 10 μM ATP or UTP was inhibited in MDA-MB-231 transfected with P2Y_2_R shRNA (Figure 2D). Moreover, P2Y_2_R activation by ATP or UTP induced MDA-MB-231 cell migration across the insert-well membrane, and this effect was blocked in P2Y_2_R knocked down MDA-MB-231 (Figure 2E).

### Nucleotides released from MDA-MB-231 cells induce the expression of AMs in ECs, increasing the adhesion of MDA-MB-231 cells to ECs and enhancing invasion through P2Y_2_R activation

**Figure 3:**
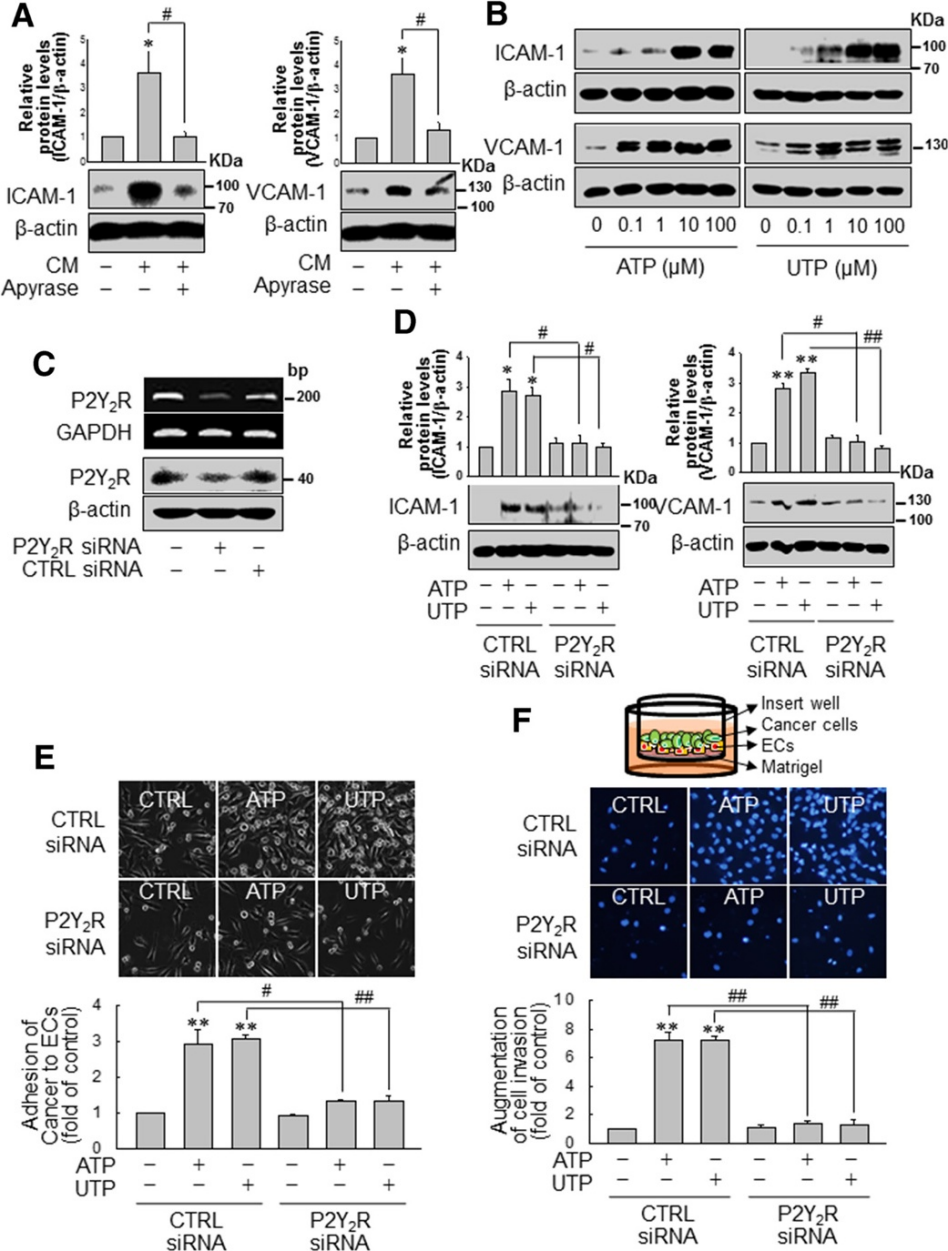
**P2Y**_**2**_**R activation by ATP or uridine 5′-triphosphate (UTP) regulated the expression of adhesion molecules in endothelial cells (ECs) and increased the adhesion and invasion of MDA-MB-231 cells in ECs. (A)** Conditioned media (CM) was obtained from MDA-MB-231 cultured in serum-free media for 16 h with or without apyrase (10 U/ml). Then, ECs were treated with CM from MDA-MB-231 for 6 h, and ICAM-1 and VCAM-1 expression was determined by western blotting. Significance compared to the control, *P <0.05; significance compared to CM, ^#^*P* <0.05. **(B)** ECs were treated with the indicated doses of ATP or UTP for 6 h, and protein expression levels were determined. **(C, D)** Control- or P2Y_2_R-siRNA-transfected ECs were treated with ATP or UTP (10 μM) for 6 h, and ICAM-1 and VCAM-1 protein expression levels were determined. Significance compared to the control, *P <0.05, **P <0.01; significance compared to ATP or UTP, ^#^*P* <0.05, ^##^*P* <0.01. **(E, F)** Control- or P2Y_2_R-siRNA-transfected MDA-MB-231 and ECs were treated with ATP or UTP (10 μM) for 6 h. Then, MDA-MB-231 were seeded onto ECs **(E)** or ECs in Matrigel-coated cell culture inserts **(F)**. After 30 minutes the remaining cell suspension (MDA-MB-231) was withdrawn, and the number of adherent cells was counted under a light microscope and quantified **(E)**. After 24 h, the numbers of cancer cells that had invaded through the EC-Matrigel-coated insert membranes were evaluated by staining with DAPI and quantified **(F)**. Significance compared to the control, **P <0.01; significance compared to ATP or UTP, ^##^*P* <0.01.

Next, we investigated the effect of ATP or UTP on the expression of AMs in ECs, the adhesion of cancer cells to ECs and cancer cell invasion through ECs. Because MDA-MB-231 released high amounts of ATP (Figure 1A), we tested whether nucleotides released from these cells could increase the expression of AMs in ECs. When ECs were treated with conditioned media (CM) from MDA-MB-231 for 6 h, ECs increased ICAM-1 and VCAM-1 expression was observed, although such expression was abolished in the presence of apyrase, an enzyme that rapidly hydrolyzes extracellular nucleotide (Figure 3A). In addition, ATP or UTP treatment dose-dependently increased ICAM-1 and VCAM-1 expression in ECs (Figure 3B). Furthermore, P2Y_2_R knockdown (Figure 3C) in ECs abolished the ATP- or UTP-mediated ICAM-1 and VCAM-1 expression in ECs (Figure 3D). Moreover, adhesion of MDA-MB-231 to ECs that were stimulated with ATP or UTP (10 μM) increased approximately 3-fold compared to non-treated cells, whereas transfection of P2Y_2_R siRNA in ECs and MDA-MB-231 markedly reduced the effect of ATP or UTP on breast cancer cell-EC adhesion (Figure 3E). Next, to investigate the role of P2Y_2_R in the invasion of MDA-MB-231 through ECs, we performed Matrigel invasion assays. MDA-MB-231 cells were pretreated with ATP or UTP for 6 h to induce AMs expression (ICAM-1 and VCAM-1) and were then harvested and seeded onto ECs-coated insert wells. ATP or UTP treatment increased the invasion of MDA-MB-231 through ECs but not through P2Y_2_R knocked down ECs (Figure 3F). Because MDA-MB-231 released much higher ATP than MCF-7 (Figure 1A), we compared the effects of nucleotides on the invasion of low or highly metastatic breast cancer cells. MDA-MB-231 showed a higher invasion than MCF-7 in basal level, which was abolished in the presence of apyrase. ATP or UTP increased the invasion of MCF-7, however which was much lower than that of MDA-MB-231 (Additional file 1: Figure S1).

### P2Y_2_R activation mediates angiogenesis through the regulation of matrix metalloproteinase (MMP) activity and vascular endothelial growth factor (VEGF) production in MDA-MB-231 cells

**Figure 4:**
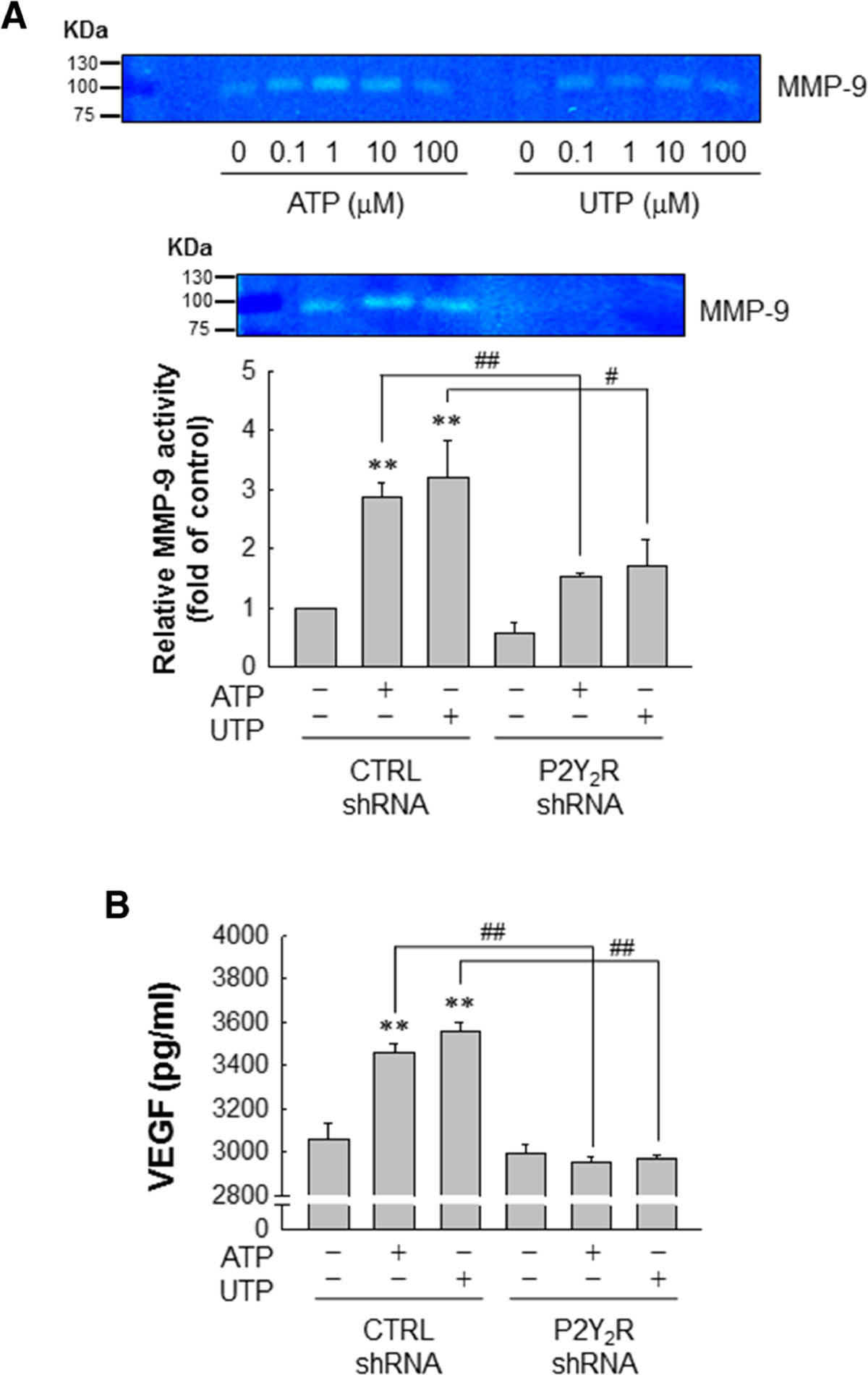
**Matrix metalloproteinase (MMP) activity and vascular endothelial growthe factor (VEGF) production in MDA-MB-231 cells was regulated by P2Y**_**2**_**R activation mediated by ATP or uridine 5′-triphosphate (UTP). (A)** MDA-MB-231 were treated with the indicated doses of ATP or UTP for 6 h. MMP gelatinase activities were measured in conditioned media (CM) as described in Methods. Control- or P2Y_2_R-shRNA-transfected MDA-MB-231 were treated with 10 μM of ATP or UTP. After 6 h, MMP gelatinase activities were determined in the CM and quantified (MMP-9; 92 KDa). Significance compared to the control, **P <0.01; significance compared to ATP or UTP, ^#^*P* <0.05, ^##^*P* <0.01. **(B)** Control- or P2Y_2_R-shRNA-transfected MDA-MB-231 were treated with 10 μM ATP or UTP for 24 h, and the concentration of VEGF from the media was determined using a quantitative VEGF ELISA. Significance compared to the control, **P <0.01; significance compared to ATP or UTP, ^##^*P* <0.01.

Because angiogenesis is a prerequisite for tumor growth and metastasis, and MMPs and VEGF are well-known to be involved in the angiogenesis, thus, we investigated the effects of P2Y_2_R activation on MMP activity and VEGF production in MDA-MB-231. ATP or UTP treatment increased MMP activity, especially that of MMP-9. In contrast, ATP or UTP (10 μM) failed to increase MMP-9 activity in MDA-MB-231 in which P2Y_2_R was knocked down (Figure 4A). Moreover, Figure 4B shows that VEGF production was significantly increased in MDA-MB-231 treated with ATP or UTP (10 μM) for 24 h compared to untreated cells. As expected, shRNA-mediated inhibition of P2Y_2_R significantly suppressed the production of VEGF induced by ATP or UTP (Figure 4B).

### ATP or UTP modulates tyrosine phosphorylation of VE-cadherin in ECs, indicating that P2Y_2_R may facilitate and increase EC permeability and breast cancer cell invasion through ECs

**Figure 5:**
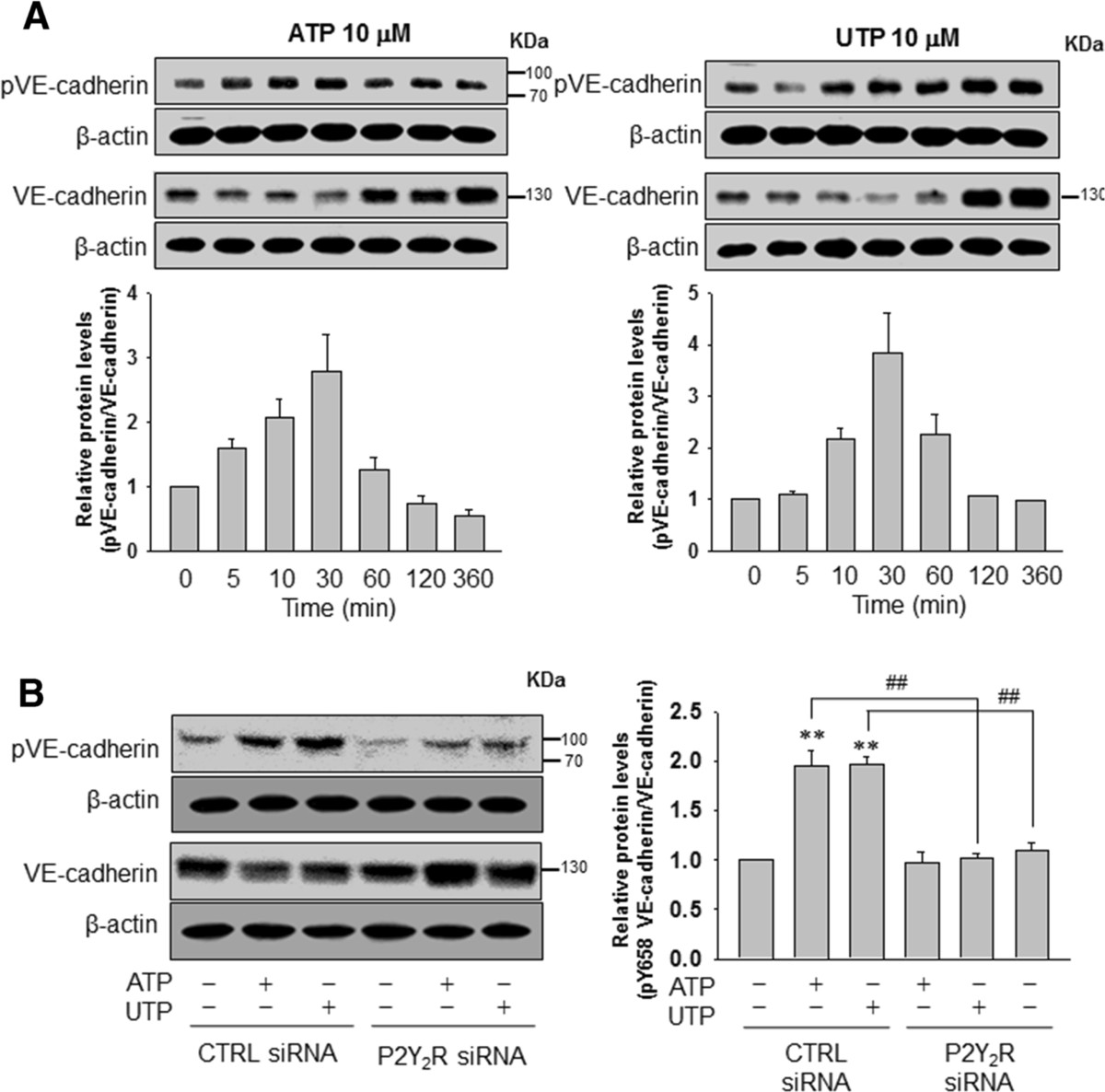
**ATP or uridine 5′-triphosphate (UTP) induced the phosphorylation of vascular endothelial (VE)-cadherin at tyrosine residue Y658 in endothelial cells (ECs). (A)** ECs were treated with ATP or UTP (10 μM) for different periods of time (5 to approximately 360 minutes). Y658-phosphorylated VE-cadherin (88 KDa), VE-cadherin (130 KDa) and β-actin protein expression levels were determined by western blotting. **(B)** ECs were transfected with control- or P2Y_2_R-siRNA, and the ECs were treated with ATP or UTP (10 μM) for 30 minutes. Protein expression levels were determined as described previously. Significance compared to the control, **P <0.01; significance compared to ATP or UTP, ^#^*P* <0.05; ^##^*P* <0.01.

Cancer cell invasion through ECs could be regulated by tyrosine phosphorylation of VE-cadherin in ECs [29], and thus, we determined whether activation of P2Y_2_R by ATP or UTP would lead to the phosphorylation of a tyrosine residue (Y658) of VE-cadherin in ECs. Our results indicated that ATP or UTP treatment increased the phosphorylation of VE-cadherin (Y658) at very early time points (5 or 10 minutes, respectively), which subsequently reached a maximum level at 30 minutes (Figure 5A) and was sustained until later times. However, ATP and UTP failed to increase the phosphorylation of Y658 VE-cadherin in P2Y_2_R-knocked-down ECs (Figure 5B).

### P2Y_2_R is involved in the tumor growth and metastasis of MDA-MB-231 breast cancer cells in an *in vivo*mouse model

**Figure 6:**
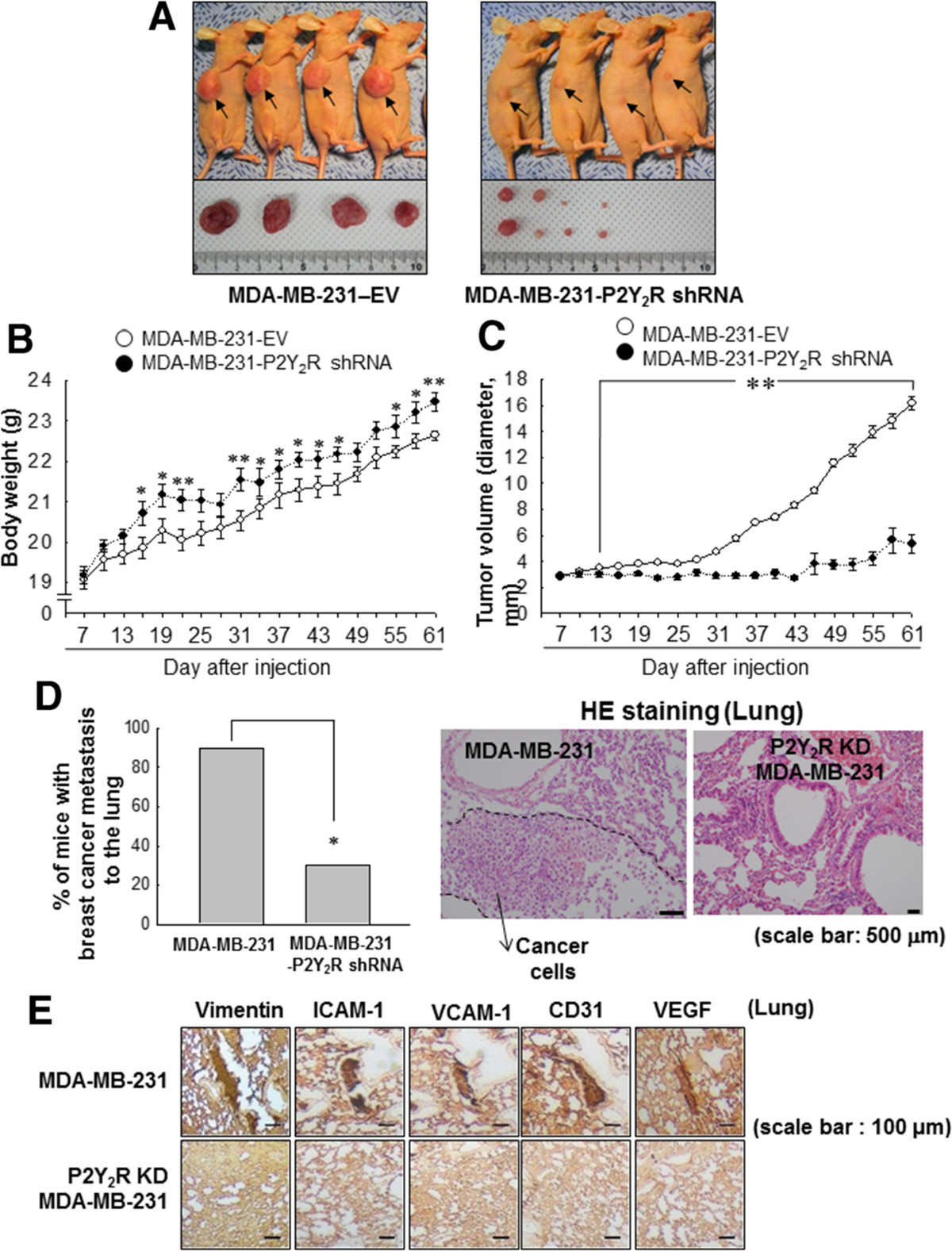
**Inhibition of P2Y**_**2**_**R reduced breast cancer cell growth and metastasis in an** ***in vivo*** **mouse model.** Athymic nude mice were divided into two groups and injected subcutaneously with empty vector-transfected MDA-MB-231 (MDA-MB-231-EV; n = 10) or P2Y_2_R-shRNA-transfected MDA-MB-231 (MDA-MB-231-P2Y_2_R-shRNA; n = 10) (5 × 10^6^ cells/100 μl of serum-free medium). **(A)** MDA-MB-231-EV-injected or MDA-MB-231-P2Y_2_R-shRNA-injected animals were sacrificed at day 60, and the tumors were extracted. Body weights **(B)** and tumor volumes **(C)** were measured every 3 days during tumor development (*P <0.05, **P <0.01 compared to the MDA-MB-231-EV-injected group). **(D)** Incidence of lung metastasis was examined in MDA-MB-231-EV- or MDA-MB-231-P2Y_2_R-shRNA-injected mice, and representative HE-stained sections are shown (*P < 0.05, compared to the MDA-MB-231-EV-injected group). **(E)** Lung tissue sections were stained with anti-vimentin, ICAM-1, VCAM-1, CD31 and VEGF antibodies.

To confirm the role of P2Y_2_R in *in vivo* tumor progression, nude mice were injected with control-shRNA-transfected MDA-MB-231 cells (MDA-MB-231-EV) or P2Y_2_R-shRNA-transfected MDA-MB-231 cells (MDA-MB-231-P2Y_2_R shRNA), and tumor volumes and body weights were measured every 3 days for 60 days. P2Y_2_R knockdown in the tumor of MDA-MB-231-P2Y_2_R-shRNA-injected mice was still functional after 60 days (Additional file 2: Figure S2). Tumor growth in mice injected with MDA-MB-231-P2Y_2_R-shRNA was significantly decreased, and their body weights were increased compared to those of mice injected with MDA-MB-231-EV by 2 weeks post-injection (Figure 6A-C). In our previous report [32] and the present study, MDA-MB-231-EV-injected mice showed a high number of cancer cells that had invaded into the lungs (9 of 10 mice), however, this was also seen in 3 of 10 mice injected with MDA-MB-231-P2Y_2_R-shRNA (Figure 6D) (*P* = 0.018, Fisher’s exact test). Additionally, we examined the lungs of tumor-bearing mice for MDA-MB-231 metastatic lesions using an antibody specific for human vimentin as described by Luga *et al.* [36]. Interestingly, vimentin expression was detected in the lungs of MDA-MB-231-EV-injected mice but not in the lungs of mice injected with MDA-MB-231-P2Y_2_R-shRNA. Furthermore, AMs (ICAM-1 and VCAM-1) and angiogenesis markers (CD31 and VEGF) expressions were also detected in the lungs of MDA-MB-231-EV-injected mice, but not in those of MDA-MB-231-P2Y_2_R-shRNA-injected mice (Figure 6E).

## Discussion

Cancer metastasis requires communication between tumor cells and ECs that culminates in the disruption of EC-EC contacts and degradation of the vascular basement membrane. However, the molecular mechanisms that facilitate such communication are not fully understood. Recently, it was reported that the tumor microenvironment affects tumor progression and the formation of metastases by mediating interactions between tumor cells, their secreted factors and the endothelium [37, 38]. Considering that tumor metastasis is the main cause of mortality in cancer patients [39], it is important to investigate the differences between highly metastatic cancer cells and low metastatic cancer cells, such as MDA-MB-231 and MCF-7, respectively, to understand how highly metastatic cancer cells communicate with ECs. It was also reported that malignant tumors trigger a strong inflammatory response and are frequently characterized by the formation of diffuse necrotic foci. Under these conditions, ATP is released from the malignant tumor and accumulates at high concentrations in the tumor interstitium to induce tumor progression. Thus, we first sought to determine the amount of ATP released from MDA-MB-231 and MCF-7. Interestingly, the highly metastatic breast cancer cell line MDA-MD-231 released significantly more ATP than the less metastatic MCF-7 cell line or normal epithelial cells or ECs, and this effect was dramatically enhanced by TNF-α. These findings are strongly supported by those of Kawai *et al.*, who also reported that the supernatant from MDA-MB-231, but not MCF-7, stimulated significantly elevated expression of ICAM-1 in ECs [40]. Although these authors identified the responsible substance from the supernatant as ATP, they did not show the exact receptor through which ATP may act but suggested that this may occur via a purinergic P2X or P2Y receptor. Among the receptors engaged by extracellular ATP (P2 receptors), P2Y_2_R is the one most overexpressed in tumor cells and mediates cell proliferation in most cancer cell types [41]. ATP also activates P2X_7_R, and this interaction is known to alter cancer cell function. However, the role of P2X_7_R in cancer is controversial; some studies have reported that P2X_7_R activation by ATP produces a trophic, growth-promoting effect [8], but according to the review by White and Burnstock [41], P2X_7_R may also decrease the number of cancer cells in contrast to P2Y_2_R. In this respect, investigating the role of P2Y_2_R and its potential involvement in cancer metastasis is important for developing effective anticancer strategies. Thus, in this study, we aimed to determine the role of P2Y_2_R, which is activated equipotently by ATP and UTP, in cancer cell metastasis, and we found that P2Y_2_R activation by ATP or UTP increased MDA-MB-231 cell proliferation and AM-mediated migration and also stimulated AM expression in ECs, resulting in increased adhesion of MDA-MB-231 to ECs. Moreover, P2Y_2_R activation by ATP or UTP increased MMP-9 activity and VEGF production in MDA-MB-231, VE-cadherin phosphorylation in ECs and MDA-MB-231 invasion through ECs.

Finally, the role of P2Y_2_R in cancer progression was confirmed using a P2Y_2_R siRNA *in vitro* transfection system (MDA-MB-231 and ECs) and an *in vivo* mice model in which animals were injected with P2Y_2_R-shRNA-transfected MDA-MB-231 cells. Interestingly, there was no significant difference in P2Y_2_R expression levels between MCF-7 and MDA-MB-231, and MCF-7 also showed an increase in (Ca^2+^)_i_. However, the transient elevation of (Ca^2+^)_i_ levels in MCF-7 were much lower than MDA-MB-231 in response to ATP or UTP (Figure 1C), suggesting the higher P2Y_2_R activity in MDA-MB-231 than MCF-7. Chadet *et al.* [42] also showed the expression and activity of P2Y_2_Rs in MCF-7 breast cancer cells promoting their migration. However, they did not show the ability of MCF-7 to invade through Matrigel and ECs. According to our data (Additional file 1: Figure S1), MDA-MB-231 showed a higher invasion than MCF-7 in basal level, which was abolished in the presence of apyrase. ATP or UTP increased the invasion of MCF-7, however which was much lower than that of MDA-MB-231. Actually, Ca^2+^ signals following UTP/ATP stimulation in MDA-MB-231 was significantly but not completely reduced by P2Y_2_R siRNA, which is possibly due to the incomplete knock-down efficiency of P2Y_2_R. Various studies have also reported that P2Y_2_R is most consistently expressed by tumor cells, however the role of P2Y_2_R on the tumor growth is controversial depending on tumor types [9–15]. Thus, our results propose that functional P2Y_2_R activation in highly metastatic breast cancer cells MDA-MB-231 mediate the signaling pathways that are involved in metastasis, and further study is needed to examine the signaling differences between MDA-MB-231 and MCF-7.

CAMs take part in intercellular and ECM interactions in cancer, and they play a pivotal role in cancer recurrence, invasiveness and the development of distant metastases. This study demonstrated that P2Y_2_R activation by ATP or UTP markedly induced ICAM-1 and VCAM-1 expression at very low doses (from 0.1 μM) in MDA-MB-231 and ECs. Furthermore, MDA-MB-231 or ECs in which P2Y_2_R was knocked down failed to induce ICAM-1 or VCAM-1 expression. In addition, the supernatant from MDA-MB-231 significantly increased ICAM-1 and VCAM-1 expression in ECs, which was diminished in the presence of apyrase, an enzyme that rapidly hydrolyzes extracellular nucleotides. Thus, these data suggest that P2Y_2_R activation by nucleotides released from MDA-MB-231 induces AMs by MDA-MB-231 and ECs, which may play an important role in cancer cell migration, cancer cell adhesion to ECs and cancer cell invasion through ECs.

The concentration of ATP in the extracellular space is the net result of release and degradation; therefore, the actual concentration of ATP released from cancer cells could be higher than the extracellular ATP concentration measured in the tumor milieu. Pellegatti *et al*. [5] measured real-time ATP concentrations within the tumor microenvironment using a chimeric plasma membrane-targeted luciferase probe *in vivo* and reported that the extracellular ATP concentration was in the hundred micromolar range in the tumor extracellular milieu, whereas it was undetectable (submicromolar) in healthy tissues. In this study, the concentration of ATP released from MDA-MB-231 was approximately 50 nM and 100 nM in the untreated condition and the TNF-α-stimulated condition, respectively. As mentioned previously, ATP or UTP was able to induce ICAM-1 and VCAM-1 expression at very low doses (100 nM) via P2Y_2_R activation, which indicates that ATP levels within the tumor microenvironment are sufficient to stimulate P2Y_2_R in MDA-MB-231 or ECs.

Endothelial permeability is one of the main factors influencing intravasation, extravasation and invasion in cancer metastasis. ECs possess several molecular mechanisms by which vascular permeability can be modulated, including the organization of adherens junctions, and in several cases, the targeting of VE-cadherin specifically. Furthermore, the phosphorylation, cleavage and internalization of VE-cadherin are thought to affect endothelial permeability [29], and VEGF and MMPs are well-known factors that mediate invasion through ECM remodeling [43, 44] and the modulation of EC permeability [45, 46]. In this study, we determined the effect of P2Y_2_R on the phosphorylation of VE-cadherin (Y658) in ECs and the MMP-9 activity and VEGF production in MDA-MB-231. According to Schumacher *et al.* [47], they also showed that ATP release from tumor cell-activated platelets activated P2Y_2_Rs on ECs to promote the extravasation of cancers at metastatic sites. However, they didn’t describe the mechanism for the tumor cell extravasation through endothelial cells. Our results showed that P2Y_2_R activation by ATP or UTP induced MMP-9 activity, VEGF production and VE-cadherin phosphorylation, and these effects of P2Y_2_R suggest that it plays an important role in promoting cancer metastasis. Taken together, our findings suggest for the first time that MDA-MB-231 highly metastatic breast cancer cells release higher levels of ATP and show higher P2Y_2_R activity in comparison to MCF-7 low metastatic breast cancer cells, and that ATP-mediated activation of P2Y_2_R plays an important role in cancer metastasis by modulating crosstalk between cancer cells and ECs.

## Conclusion

**Figure 7:**
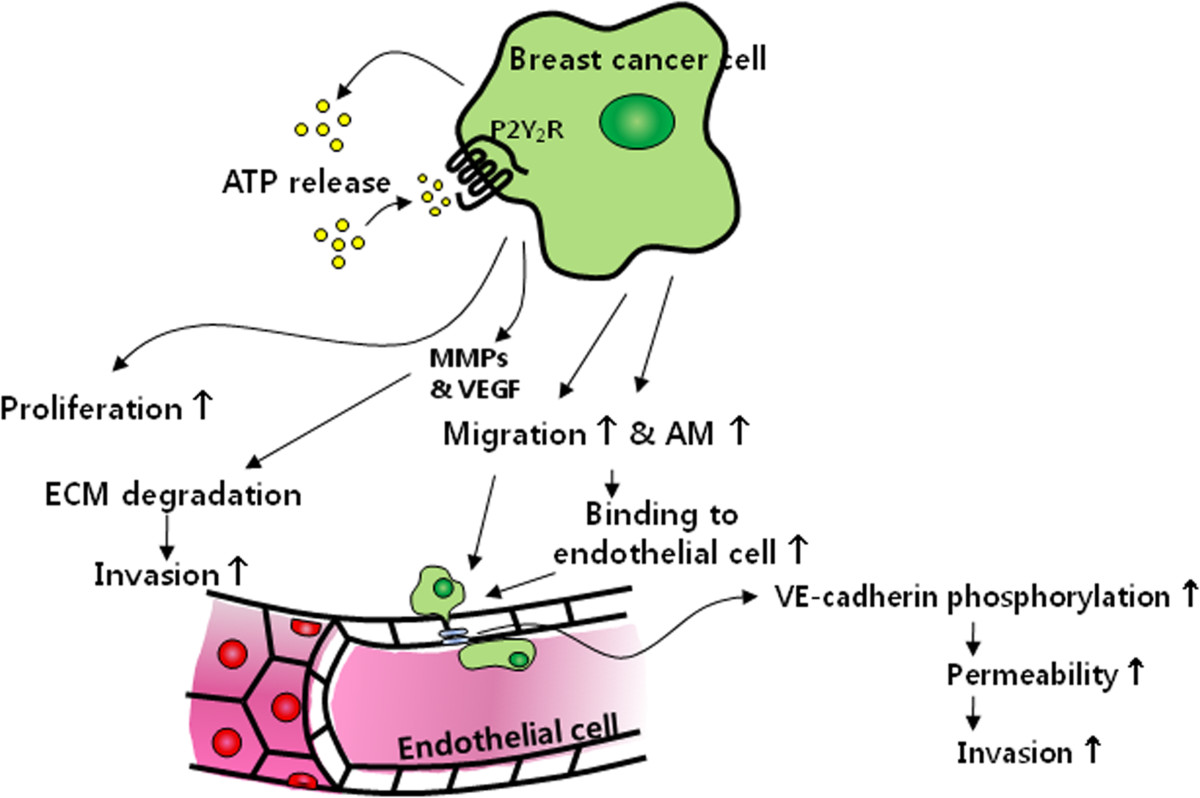
**Schematic representation of the proposed role of P2Y**_**2**_ **R in MDA-MB-231 breast cancer cell progression.**

MDA-MB-231 highly metastatic breast cancer cells release higher levels of ATP in comparison to MCF-7 low metastatic breast cancer cells, and ATP-mediated activation of P2Y_2_R plays an important role in cancer metastasis by modulating crosstalk between cancer cells and ECs (Figure 7).

## Electronic supplementary material

Additional file 1: Figure S1: **A**) ECs were added to Matrigel-coated 24-well cell culture inserts. MCF-7 and MDA-MB-231 cells were treated with apyrase (10 U/ml), ATP or UTP (10 μM) for 6 h and seeded onto ECs in Matrigel-coated cell culture inserts. After 24 h, the numbers of cancer cells that had invaded through the EC-Matrigel-coated insert membranes were evaluated by staining with DAPI. **B-C**) The number of cells that invaded through the membrane was quantified by counting cells under a fluorescence microscope. Values represent the means ± SEM of 3 independent experiments (B, **P < 0.01; C, significance compared to the control of MCF-7, **P < 0.01; significance compared to the control of MDA-MB-231, ^##^*P* < 0.01). (TIFF 985 KB)

Additional file 2: Figure S2: Tumor tissue sections from MDA-MB-231-EV- and MDA-MB-231-P2Y_2_R-shRNA-injected mice were stained with H&E and anti-P2Y_2_R antibody (400x or 200x magnification). (TIFF 4 MB)

Below are the links to the authors’ original submitted files for images.

Authors’ original file for figure 1

Authors’ original file for figure 2

Authors’ original file for figure 3

Authors’ original file for figure 4

Authors’ original file for figure 5

Authors’ original file for figure 6

Authors’ original file for figure 7

## Abbreviations

AM: adhesion molecules
ATP: adenosine triphosphate
bp: base pairs
CM: conditioned media
CTRL: control
DAPI: 4′, 6-diamidino-2-phenylindole dihydrochloride
DMEM: Dulbecco’s modified Eagle’s medium
EC: endothelial cell
ECM: extracellular matrix
ELISA: enzyme-linked immunosorbent assay
ER: estrogen receptor
FBS: fetal bovine serum
GAPDH: glyceraldehyde-3-phosphate dehydrogenase
H&E: hematoxylin and eosin
ICAM-1: intercellular adhesion molecule-1
MMP: matrix metalloproteinase
P2Y_2_R: P2Y_2_ receptor
PBS: phosphate-buffered saline
RT-PCR: reverse transcription-polymerase chain reaction
SDS-PAGE: sodium dodecyl sulfate-polyacrylamide gel electrophoresis
shRNA: small hairpin RNA
siRNA: small interferin RNA
TNF: tumor necrosis factor
UTP: uridine 5′-triphosphate
VCAM-1: vascular cell adhesion molecule-1
VE-cadherin: vascular endothelial cadherin
VEGF: vascular endothelial growth factor.
