# Supplementary figures and images for: P2Y2receptor activation by nucleotides released from highly metastatic breast cancer cells increases tumor growth and invasion via crosstalk with endothelial cells
Source: Breast Cancer Res. 2014 Aug 26;16:R77. doi: 10.1186/bcr3694 (PMC4406012; doi:10.1186/bcr3694)

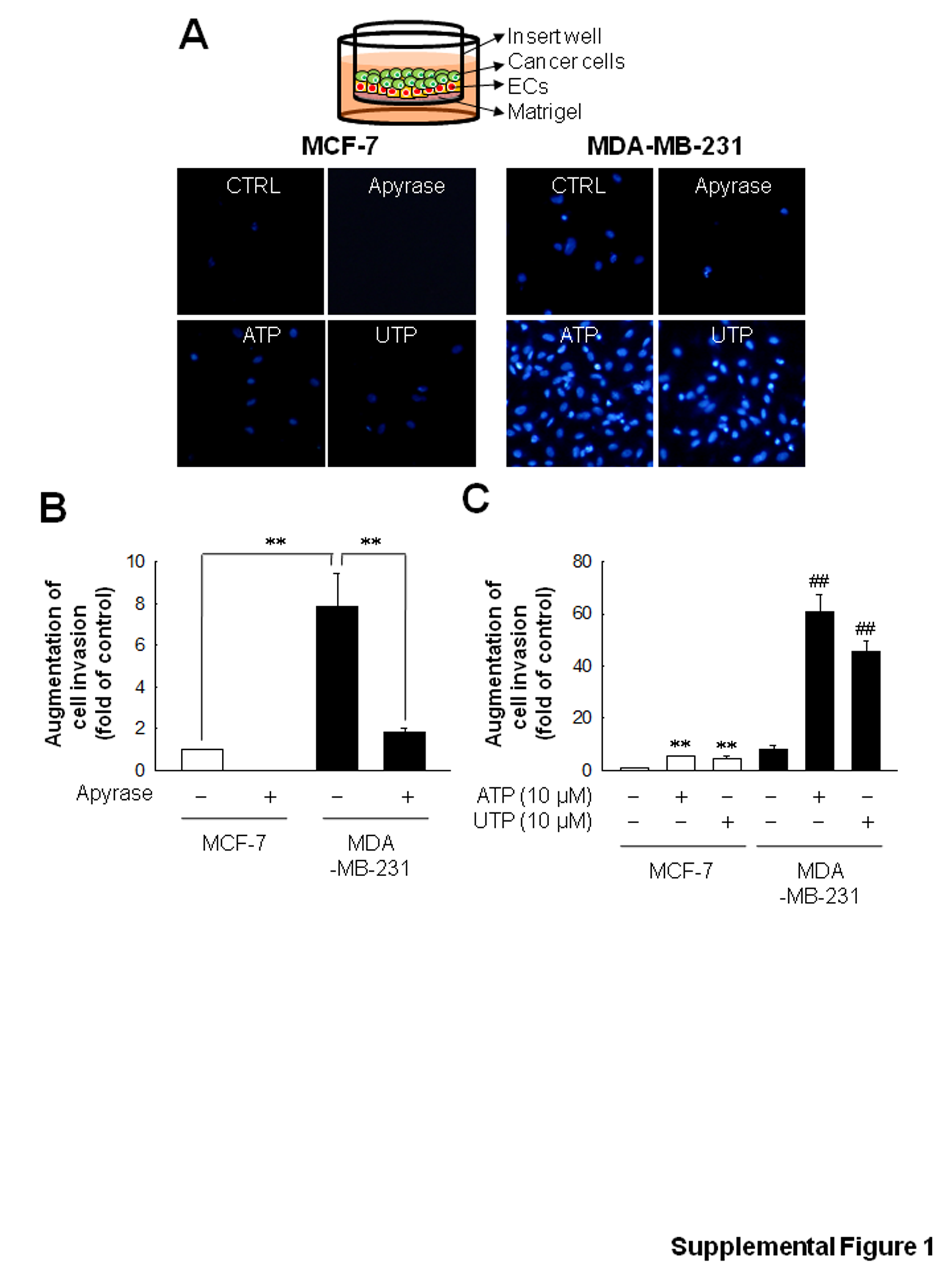

Supplement: Supplementary file 1 — Additional file 1: Figure S1: A) ECs were added to Matrigel-coated 24-well cell culture inserts. MCF-7 and MDA-MB-231 cells were treated with apyrase (10 U/ml), ATP or UTP (10 μM) for 6 h and seeded onto ECs in Matrigel-coated cell culture inserts. After 24 h, the numbers of cancer cells that had invaded through the EC-Matrigel-coated insert membranes were evaluated by staining with DAPI. B-C) The number of cells that invaded through the membrane was quantified by counting cells under a fluorescence microscope. Values represent the means ± SEM of 3 independent experiments (B, **P < 0.01; C, significance compared to the control of MCF-7, **P < 0.01; significance compared to the control of MDA-MB-231, ##P < 0.01). (TIFF 985 KB) [file 13058_2014_3466_MOESM1_ESM.tiff]

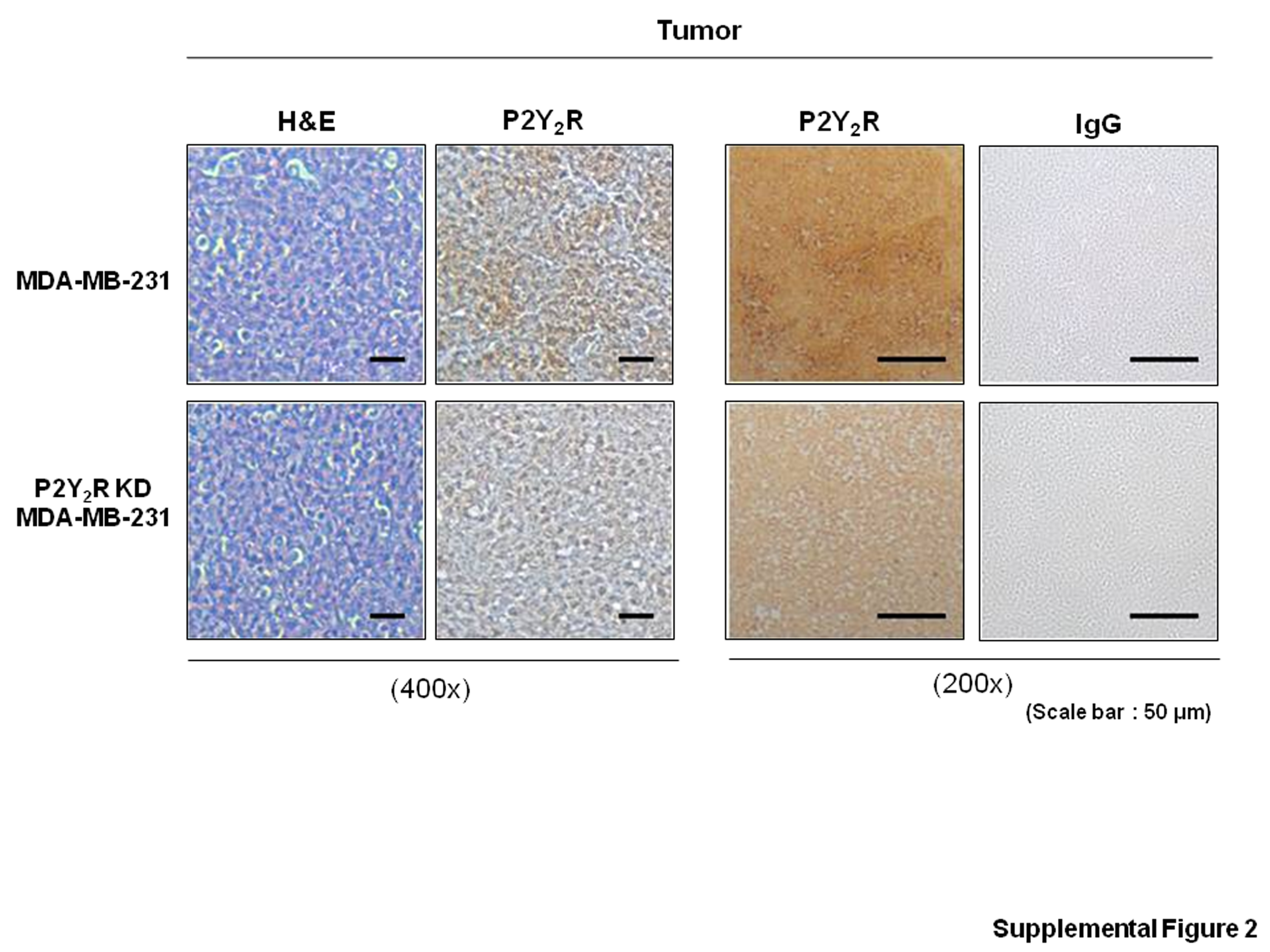

Supplement: Supplementary file 2 — Additional file 2: Figure S2: Tumor tissue sections from MDA-MB-231-EV- and MDA-MB-231-P2Y2R-shRNA-injected mice were stained with H&E and anti-P2Y2R antibody (400x or 200x magnification). (TIFF 4 MB) [file 13058_2014_3466_MOESM2_ESM.tiff]

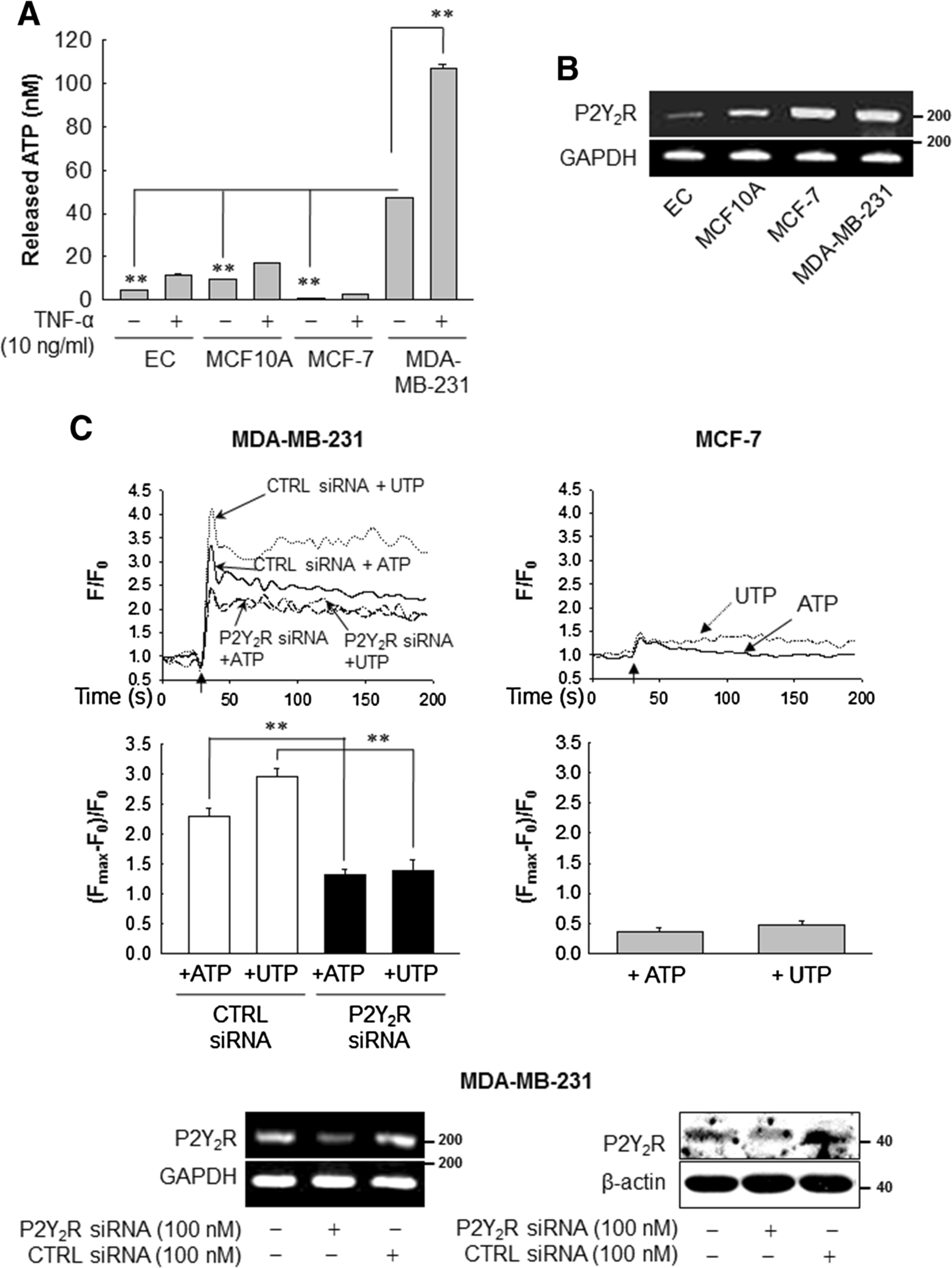

Supplement: Supplementary file 3 — Authors’ original file for figure 1 [file 13058_2014_3466_MOESM3_ESM.tif]

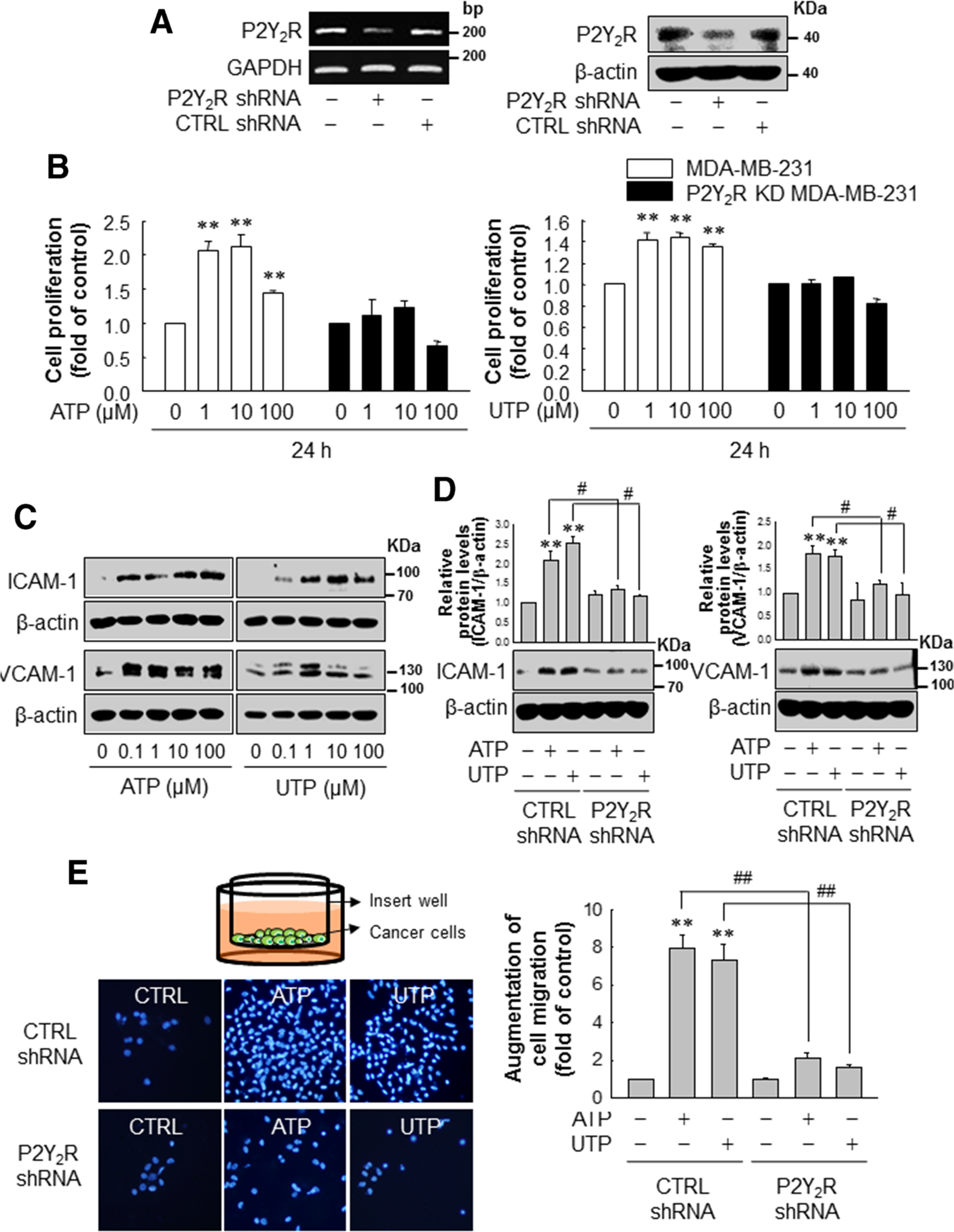

Supplement: Supplementary file 4 — Authors’ original file for figure 2 [file 13058_2014_3466_MOESM4_ESM.tif]

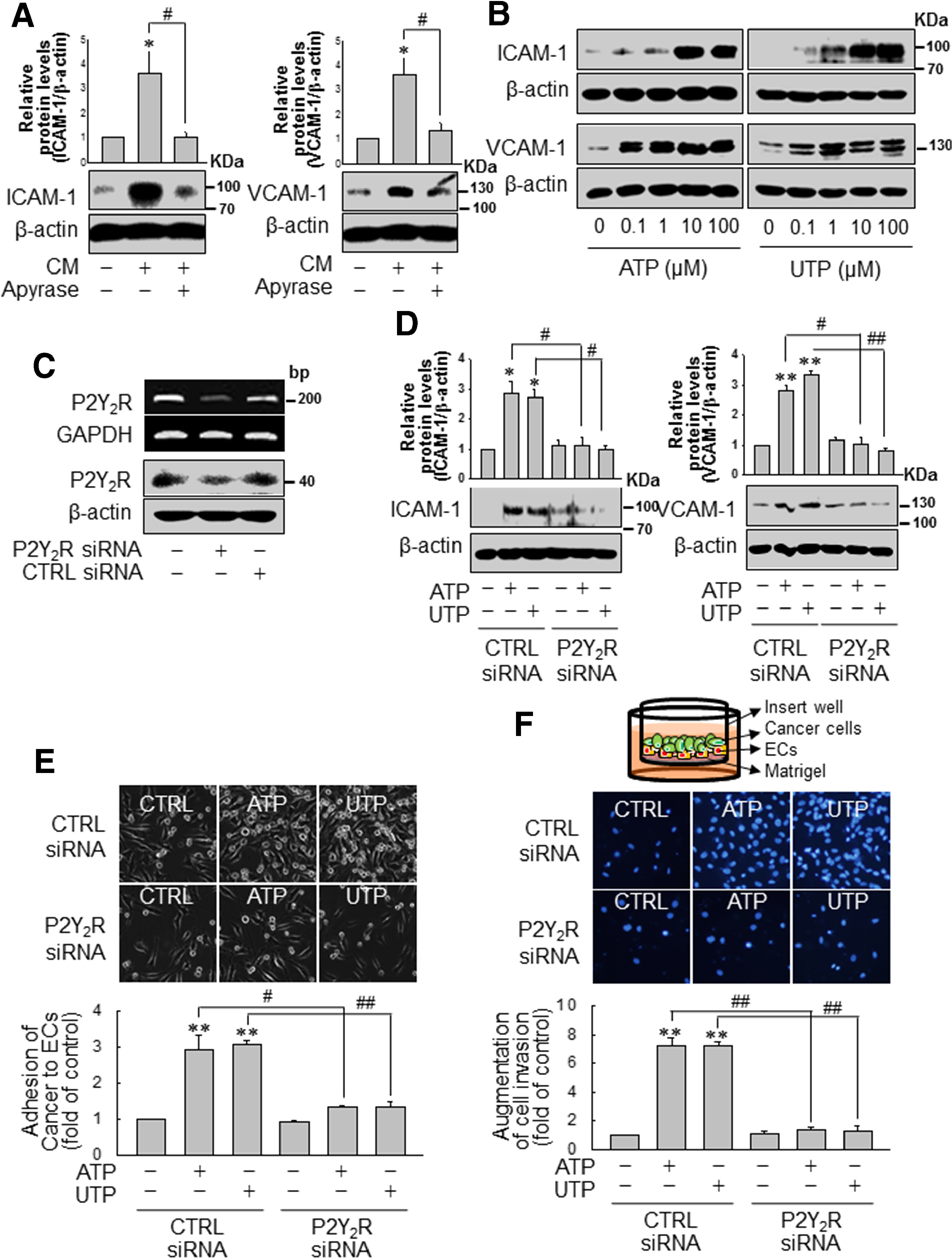

Supplement: Supplementary file 5 — Authors’ original file for figure 3 [file 13058_2014_3466_MOESM5_ESM.tif]

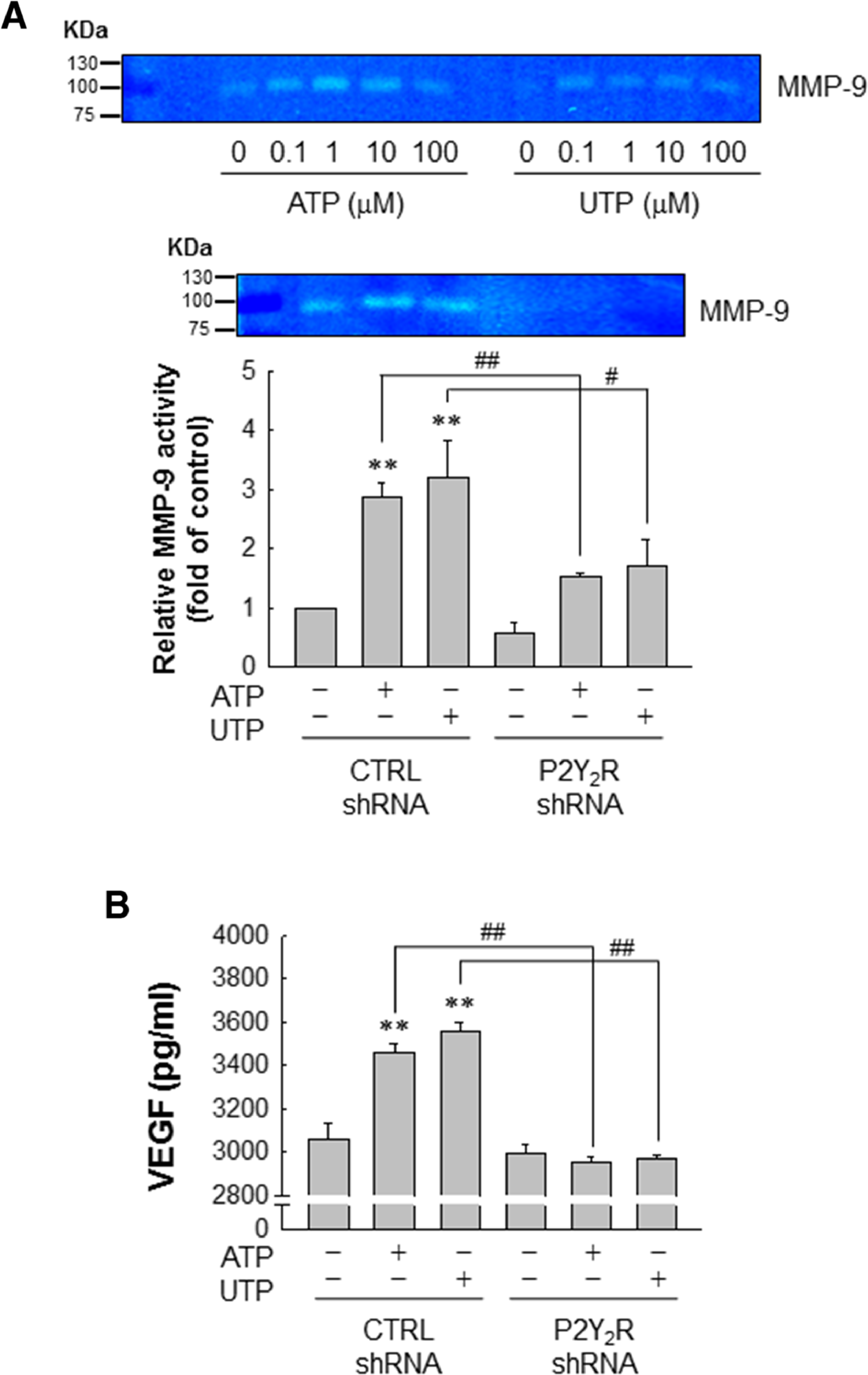

Supplement: Supplementary file 6 — Authors’ original file for figure 4 [file 13058_2014_3466_MOESM6_ESM.tif]

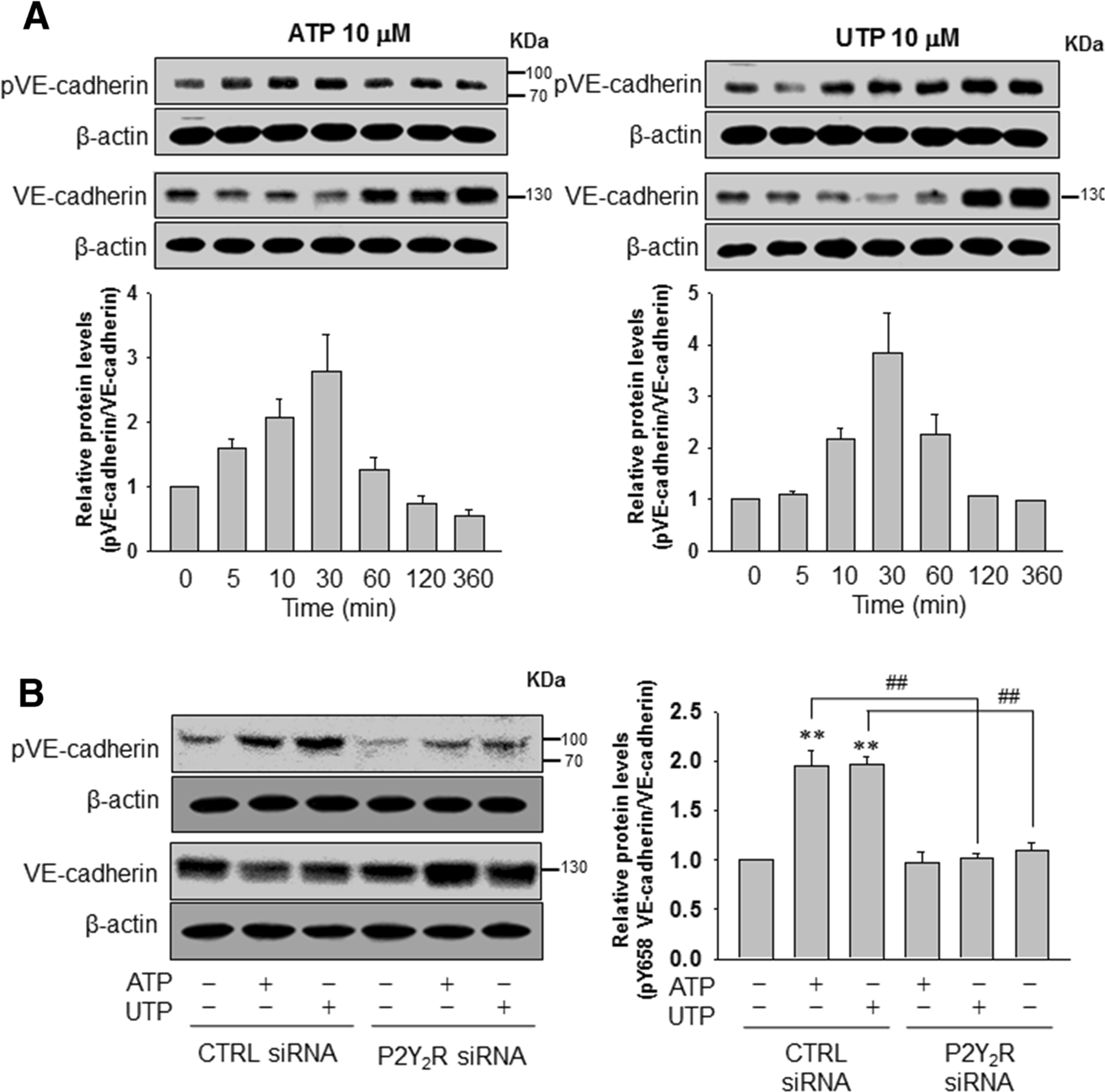

Supplement: Supplementary file 7 — Authors’ original file for figure 5 [file 13058_2014_3466_MOESM7_ESM.tiff]

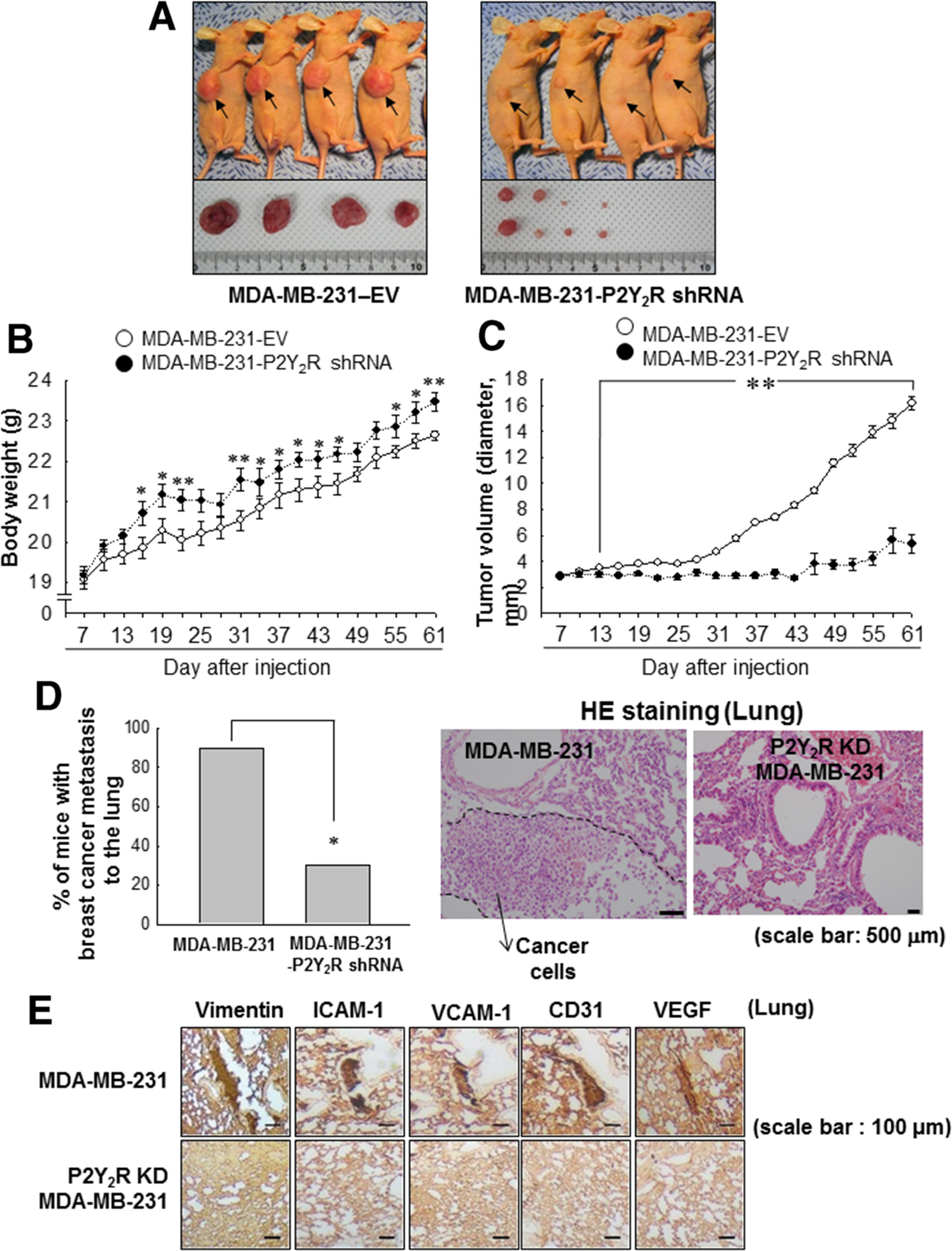

Supplement: Supplementary file 8 — Authors’ original file for figure 6 [file 13058_2014_3466_MOESM8_ESM.tiff]

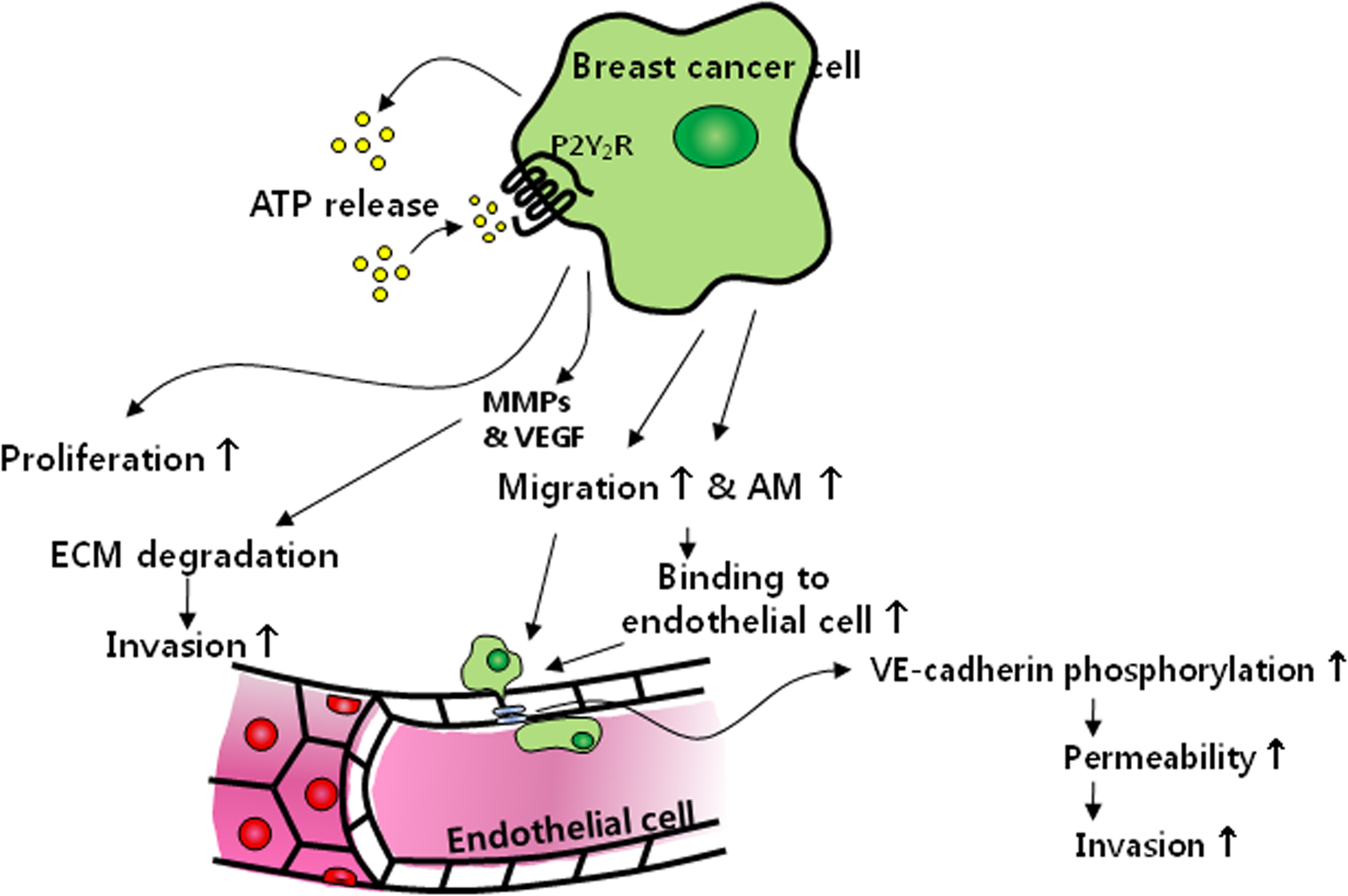

Supplement: Supplementary file 9 — Authors’ original file for figure 7 [file 13058_2014_3466_MOESM9_ESM.tif]
